# Cancer-associated fibroblasts gene signature: a novel approach to survival prediction and immunotherapy guidance in colon cancer

**DOI:** 10.3389/fimmu.2025.1532306

**Published:** 2025-04-08

**Authors:** Wenbing Zhang, Chi Yang, Ye Lu, Chenling Tang, Mengyu Zhao, Zhaohui Wang, Jidong Gao, Shuangjiu Hu, Zhihua Chen

**Affiliations:** ^1^ Department of General Surgery, Anqing First People’s Hospital of Anhui Medical University, Anqing, China; ^2^ Department of General Surgery, QingPu Branch of Zhongshan Hospital Affiliated to Fudan University, QingPu District Central Hospital Shanghai, No. 1158, Gong Yuan Dong Road, Shanghai, China; ^3^ Department of Breast Surgical Oncology, National Cancer Center/National Clinical Research Center for Cancer/Cancer Hospital, Chinese Academy of Medical Sciences and Peking Union Medical College, Beijing, China; ^4^ Department of Breast Surgical Oncology, National Cancer Center/National Clinical Research Center for Cancer/Cancer Hospital & Shenzhen Hospital, Chinese Academy of Medical Sciences and Peking Union Medical College, Shenzhen, China; ^5^ The First People’s Hospital of Taicang City, Taicang Affiliated Hospital of Soochow University, Suzhou, Jiangsu, China

**Keywords:** colon adenocarcinoma, cancer-associated fibroblasts, signature, tumor immune microenvironment, MAN1B1

## Abstract

**Background:**

Fibroblasts can regulate tumour development by secreting various factors. For COAD survival prediction and CAFs-based treatment recommendations, it is critical to comprehend the heterogeneity of CAFs and find biomarkers.

**Methods:**

We identified fibroblast-associated specific marker genes in colon adenocarcinoma by single-cell sequencing analysis. A fibroblasts-related gene signature was developed, and colon adenocarcinoma patients were classified into high-risk and low-risk cohorts based on the median risk score. Additionally, the impact of these risk categories on the tumor microenvironment was evaluated. The ability of CAFGs signature to assess prognosis and guide treatment was validated using external cohorts. Ultimately, we verified MAN1B1 expression and function through *in vitro* assays.

**Results:**

Relying on the bulk RNA-seq and scRNA-seq data study, we created a predictive profile with 11 CAFGs. The profile effectively differentiated survival differences among cohorts of colon adenocarcinoma patients. The nomogram further effectively predicted the prognosis of COAD patients, with low-risk patients having a better prognosis. A higher immune infiltration rate and lower IC50 values of anticancer drugs were significant in the high-risk group. In cellular experiments, Following MAN1B1 knockdown, in cell assays, the colony formation, migration, and invasion ability of HCT116 and HT29 cell lines decreased.

**Conclusion:**

Our CAFG signature provides important insights into the role of CAF cells in influencing COAD prognosis. It may also serve as a guide for selecting immunotherapy options and predicting chemotherapy responses in COAD patients.

## 1 Introduction

Colon adenocarcinoma (COAD) is a heterogeneous neoplastic disease characterized by diverse stromal cell population infiltration. The progression of COAD is driven by the accumulation of oncogenic mutations and the dynamic interactions within the tumor microenvironment (TME) (1, 2). The TME, comprising non-epithelial cells and extracellular matrix components, plays a critical role in tumor development and therapy resistance. However, the traditional pathological staging system often fails to accurately predict patient outcomes, highlighting the need for more reliable prognostic models (3). Such models are essential for assessing patient risk drug sensitivity and guiding personalized immunotherapy and chemotherapy regimens.

TME includes a variety of non-cancerous cells, with cancer-associated fibroblasts (CAFs) being the most important and common type (4). CAFs can regulate blood vessel production and cell metabolism, subsequently driving tumors’ onset and transfer (5, 6). CAFs promote tumor immunosuppression through interactions with immune cells in the tumor immune microenvironment (7). Despite their importance, CAFs exhibit considerable heterogeneity, which is reflected in the diversity of their subpopulation markers. Commonly used markers such as αSMA and FAP are not exclusive to CAFs, as they are also expressed in pericytes and fibroblastic reticular cells (8, 9). This underscores the need to identify specific markers for CAFs to enable targeted therapeutic strategies for COAD.

Recent advances in single-cell RNA sequencing (scRNA-seq) have provided new insights into the heterogeneity of CAFs across various cancers, revealing potential therapeutic targets. By integrating single-cell and bulk RNA-seq analyses, we identified 11 CAF-related genes that may serve as promising targets for COAD treatment. Further *in vitro* experiments confirmed the expression of MAN1B1 in COAD cells, suggesting its potential as a therapeutic target. This study explores the functional roles of these genes and their implications for COAD therapy.

## 2 Materials and methods

### 2.1 Data collection

We selected 23 tumour samples by downloading scRNA-seq data of GSE132465 via the GEO database. RNA-seq data in FPKM format and survival information for 430 TCGA-COAD cases were retrieved from the UCSC Xena platform (https://gdc.xenahubs.net). The normalized FPKM values were converted into TPM and further transformed using log2 (TPM+1) conversion. We also obtained normalized gene expression profiles and clinical details from the GEO database of 177 cases in the GSE17536 dataset and 171 cases in the GSE159216 dataset, averaging the values if a gene matched multiple probes.

### 2.2 Single-cell RNA-sequence analysis

The ‘Seurat’ toolkit (version 4.3.0) in R was used to standardize the scRNA-seq data’s downstream processing (10). For the scRNA-seq dataset, each gene must be expressed in at least three cells, with no less than 200 genes per cell. Furthermore, the amount of mitochondria was kept to less than 14%. The LogNormalize method was used for data normalization. After performing PCA, we employed UMAP, a non-linear dimensionality reduction technique. Subsequently, clustering was conducted using the ‘FindNeighbors’ function with a dimensionality parameter set to 1, followed by the ‘FindClusters’ function with parameters dim set to 20 and resolution set to 0.2 (11). Subsequently, we identified the marker genes unique to each cluster by utilizing the ‘FindAllMarkers’ function, setting a threshold of an absolute log2 fold change (FC) of at least 0.5 and requiring a minimum cell population percentage of 0.25 (minpct = 0.25) for each cluster. Following this, we employed the ‘SingleR’ tool (version 1.10.0) to annotate the cell types based on the identified markers (12).

### 2.3 GO and KEGG pathway examination

To investigate the biological functions and pathways associated with CAFs-related key genes, we utilized the R tools org.Hs.eg.db (version 3.15.0) and clusterProfiler (version 4.9.0). GO functional enrichment evaluation was performed to identify differences and similarities across BP (Biological Process), CC (Cellular Component), and MF (Molecular Function) categories. Furthermore, an enrichment analysis of KEGG pathways was carried out to highlight the most prevalent pathways.

### 2.4 Creating and validating a predictive gene signature correlated with CAFs

We developed a prognostic model for COAD by identifying CAF marker genes from scRNA-seq data. We identified genes significantly correlated with overall survival (OS) through univariate Cox regression (P<0.05). To refine the model, we employed LASSO Cox regression with the glmnet package (version 4.1-6)and followed with multivariate Cox regression (13, 14). The risk score was calculated as the aggregate of the products obtained by multiplying gene expressions by their respective coefficients. We employed the timeROC package (version 0.4) to evaluate the model’s predictive accuracy and further validated its performance in separate cohorts.

### 2.5 Development of nomogram

We initially conducted univariate and multivariate Cox regression analyses on clinical and risk factors to develop a nomogram tool for clinical use. Factors with p<0.05 in the multivariate Cox analysis were selected to generate the column-line diagram for predicting COAD prognosis with the rms package (version 6.5-0). The final nomogram was developed based on CAF characteristics, M stage, and patient age. Its predictive performance and accuracy were then assessed through ROC and calibration curves to confirm reliability.

### 2.6 Immune infiltration analysis

The ESTIMATE technique was used to assess stromal and immune cell infiltration levels. RNA sequencing data from the TCGA-COAD cohort were analyzed using the ESTIMATE computational method to generate stromal, immune, estimate, and tumor purity scores. Wilcoxon tests were conducted to compare these scores across different risk groups. Fibroblast infiltration levels were determined through the Microenvironmental Cell Population Counting (MCP-counter) algorithm, utilizing the MCP-counter package (version 1.2-0) (15). The infiltration levels of 28 immune cell types were measured through single-sample Gene Set enrichment analysis (ssGSEA) (16). To evaluate the disparities in immune checkpoint blockade responses between the two groups, we employed the ‘ggpubr’ software package (version 0.6.0).

### 2.7 Identification of CAFs relevant mutations and analysis of drug sensitivity

Somatic mutation information of COAD patients was obtained from the TCGA repository. For each person, the rates of genetic mutations and the lengths of exons were determined. To compare mutations across various risk categories, waterfall plots were created utilizing the ‘maftools’ R package and visual representations of tumor mutational burden (TMB) values. The Wilcoxon test assessed the differences in TMB values between these categories. K-M analysis was used to examine OS variations between the two groups. Additionally, the chemotherapy response of COAD individuals was analyzed using the Genomics of Drug Sensitivity in Cancer (GDSC) database (17). The “pRRophetic” package (18) was employed to estimate the 50% maximal inhibitory concentration (IC50) to evaluate chemotherapy sensitivity.

### 2.8 TIMER database analysis

MAN1B1 expression in COAD and adjacent normal tissues were examined through the TIMER database, which includes 10,897 samples from 32 distinct cancer types. The DiffExp module was employed to evaluate MAN1B1 expression in a pan-cancer context.

### 2.9 Cell culture, transfection and reverse transcription-quantitative PCR

The COAD cell lines, HCT116 and HT29, were acquired from the Chinese Cell Culture Collection and cultured in RPMI 1640 medium supplemented with 10% fetal bovine serum (FBS) and 100 units per milliliter of penicillin-streptomycin. MAN1B1 knockdown was confirmed by qRT-PCR. Cells treated with NC, siMAN1B1-1, and siMAN1B1-2 to inhibit MAN1B1 expression were used in subsequent experiments (19). **Supplementary Table S1** contains the primer sequences.

### 2.10 Colony formation assay

Four hundred transfected cells were cultured in 6-well plates for approximately 14 days. The samples underwent fixation with 4% paraformaldehyde for 15 minutes, which was succeeded by a 20-minute staining process using Crystal Violet (Solarbio, China). Following the staining, the cells were allowed to air-dry at room temperature, and then the cell count in each well was determined.

### 2.11 Wound healing assay

After plating the transfected cells into 6-well plates, then cultured in a cell incubator until achieving 95% confluence. A straight line was then drawn using a sterile 200 μL pipette tip. Then, carefully wash away any detached cells and debris with PBS. Subsequently, the cells were moved into a cell growth medium devoid of serum. Finally, Image J was used to estimate the breadth of the scratches after pictures were taken at the same location at 0 and 48 hours.

### 2.12 Transwell experiment

Transwell experiments were employed to evaluate cell invasion and migration. Treated cells were placed in the upper chamber in 200 μl of medium without serum, with 5×10^4^ cells per well. The upper chamber was coated with Matrigel solution to evaluate invasive and migratory capacity, while the lower chamber contained 700 μl of complete medium. Photographs and counts of the successfully moving cells were taken.

### 2.13 Statistical analysis

The data are presented as the mean ± standard deviation. To evaluate the differences between groups, a Student’s t-test was applied, and all experiments were conducted at least three times. The statistical analyses used GraphPad Prism version 9.1.1 and R version 4.1.1.

## 3 Result

### 3.1 Single cell RNA-seq analysis

**Figure 1:**
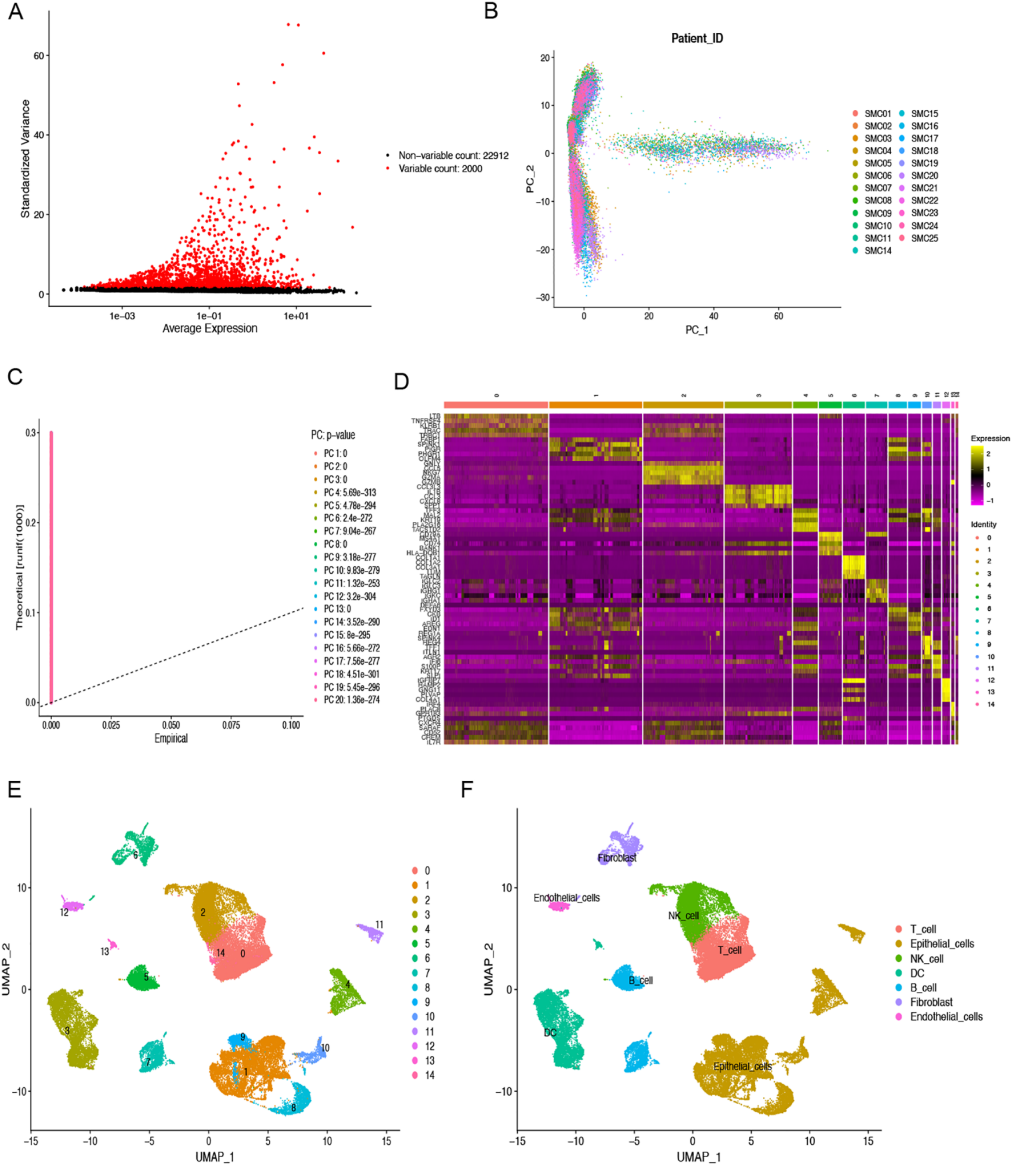
scRNA-seq analysis to identify fibroblasts marker genes. **(A)** The top 2000 highly variable genes are highlighted in red dots. **(B)** PCA was used to decrease dimensionality. **(C)** The top 20 PCs were identified with the P-value < 0.05. **(D)** The heatmap indicated the relative gene expression of 15 clusters. Genes with high expression are depicted in yellow, whereas genes with low expression are highlighted in purple. **(E)** Fifteen clusters were visualized using the UMAP technique. **(F)** Cell subpopulations identified by marker genes. Different color areas represent different cells.

Quality control preprocessing of scRNA-seq data was conducted according to appropriate metrics, and the quality control results are shown in **Supplementary Figure S1**. 47285. High quality cell samples were isolated from 23 COAD tissues screened for subsequent further examination. Following data normalization, we chose the 2000 most variable genes (**Figure 1A**). Dimensionality reduction was executed employing the PCA technique (**Figure 1B**), and the 20 most significant PCs with p< 0.05 were selected for additional examination (**Figure 1C**). We successfully classified the cells into 15 distinct clusters based on the top 20 major components and identified 5356 differentially expressed marker genes across these clusters, which are listed in **Supplementary Table S2**. The heatmap illustrates the expression values of the five most significant marker genes within every cluster (**Figure 1D**). The IMAP technique also represented the multi-dimensional scRNA sequencing data (**Figure 1E**). We annotated the cell subpopulations using the “SingleR” package as recognized cell types (**Figure 1F**). The main cell categories include T cells, epithelial cells, NK cells, B cells, DCs, fibroblasts, and endothelial cells, with cluster 6 identified as the fibroblasts subpopulation. Finally, we identified 1025 significant expression marker genes of COAD-associated fibroblasts based on a threshold adjPval < 0.05.

### 3.2 Functional assessment of GO and KEGG of fibroblasts-associated genes

**Figure 2:**
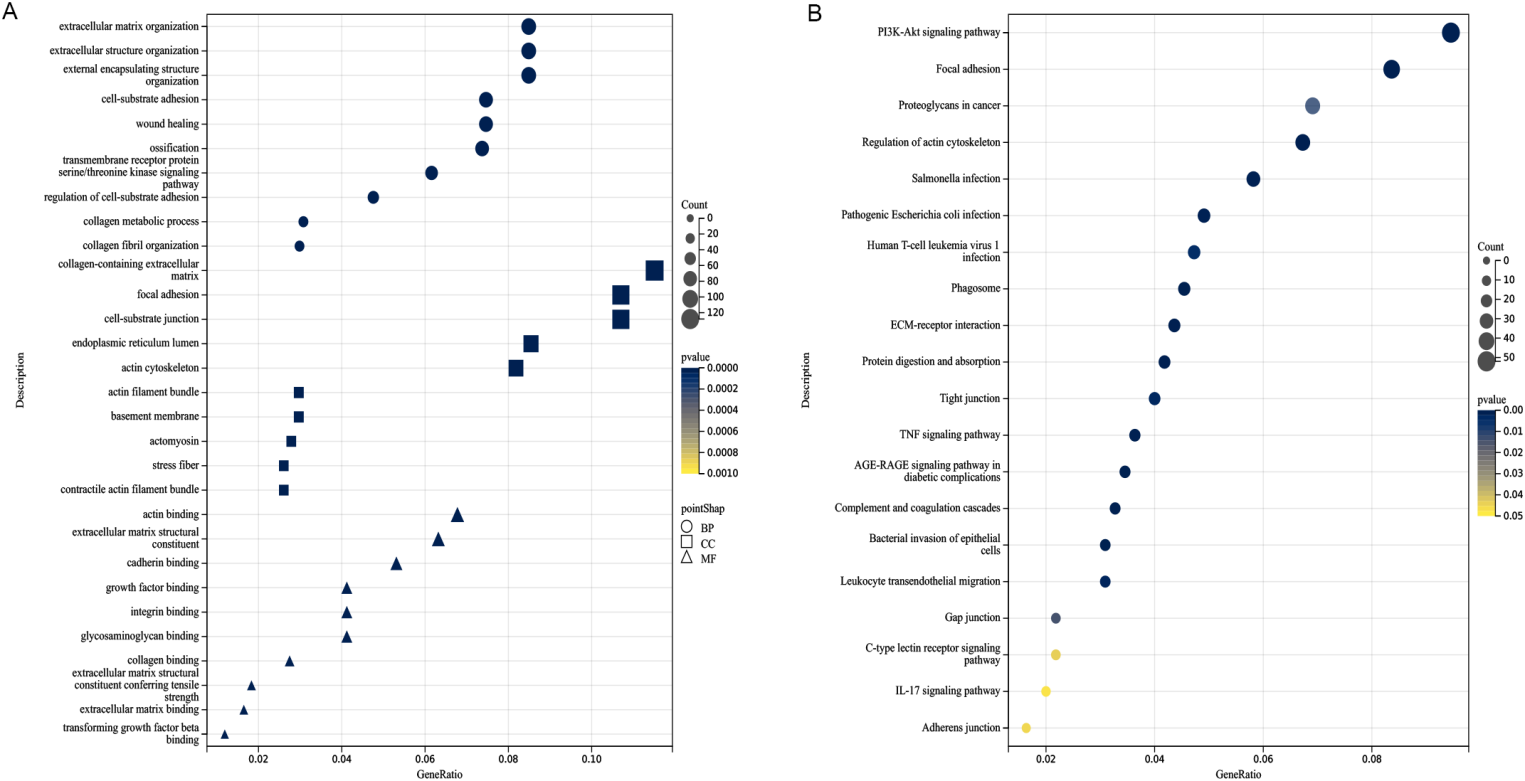
Analysis of functional enrichment. **(A)** Function enrichment analysis based on BP, CC, and MF, three different viewpoints. **(B)** The top 20 pathways of KEGG analysis. The darker the color, the smaller the P value, and the larger the shape, the larger the number.

GO and KEGG functional assessments were conducted to investigate the functions and pathways of fibroblasts-associated genes. **Figure 2A** displays the 10 most significantly enriched GO terms. For BP, the enriched terms included “extracellular structural organization,” “extracellular matrix organization,” and “external encapsulating structure organization.” In the CC category, genes were enriched in “collagen-containing extracellular matrix,” “focal adhesion,” and “cell-substrate junction.” MF terms were primarily associated with “actin binding,” “extracellular matrix structural constituent,” and “cadherin binding.” Additionally, the top 20 enriched KEGG pathways were displayed in **Figure 2B**, which included pathways such as “PI3K-Akt signaling pathway,” “focal adhesion,” “proteoglycans in cancer,” “regulation of actin cytoskeleton,” “ECM-receptor interaction,” “protein digestion and absorption,” “tight junction,” “adherens junction,” “leukocyte transendothelial migration,” and “gap junction.”

### 3.3 CAFGs signature construction and verification

**Figure 3:**
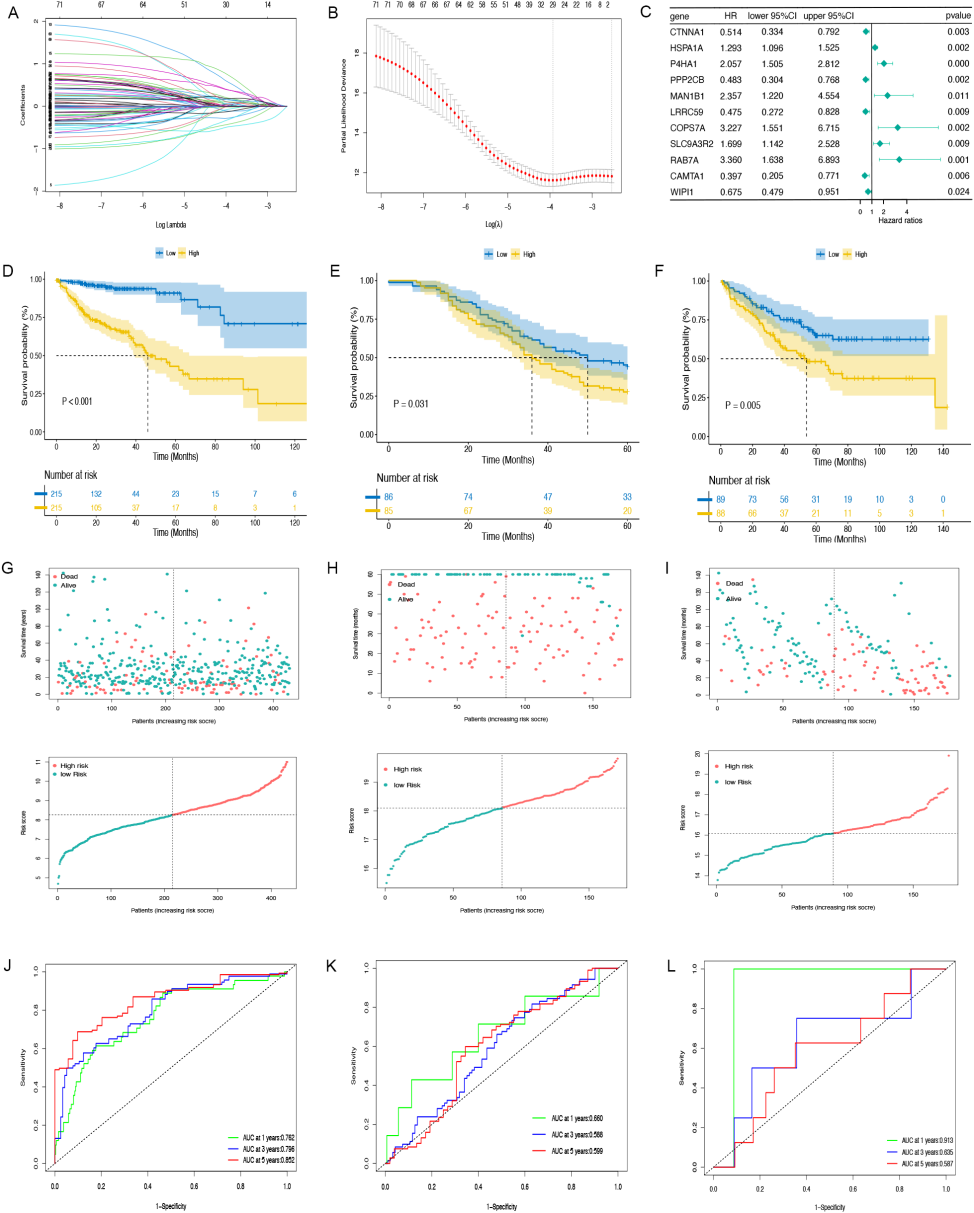
The prognostic model is constructed and validated. **(A, B)** LASSO regression analysis. **(C)** Multivariate Cox regression results are plotted in a forest. **(D–F)** The Kaplan-Meier curves in TCGA-COAD, GSE159216 and GSE17536 cohorts. **(G–I)** Distribution of CAFGs risk score and scatter plot of the OS of each patient in TCGA-COAD, GSE159216 and GSE17536 cohorts, respectively. **(J–L)** The AUC at 1-, 3-, and 5-years of prognostic models in TCGA-COAD, GSE159216 and GSE17536 cohorts.

A Cox proportional hazards analysis was performed on CAF marker genes in the TCGA-COAD dataset, identifying 144 genes with P<0.05. Following this, LASSO Cox regression analysis (**Figures 3A, B**) was conducted on the genes, resulting in the selection of 11 prognostic genes with significant non-zero coefficients (**Figure 3C**). Risk Score =(-0.66451*CTNNA1 expression)+(0.25692*HSPA1A expression)+(0.72129*P4HA1 expression)+(-0.72811*PPP2CB expression)+(0.85761 *MAN1B1 expression)+(-0.74474*LRRC59 expression)+(1.17154*CCPS7A expression)+(0.52996*SLC9A3R2 expression)+(1.21188*RAB7A expression) +(-0.92288*CAMTA1 expression)+(-0.39307*WIPI1 expression). Among the 11 identified prognostic genes, six (HSPA1A, P4HA1, MAN1B1, CCPS7A, SLC9A3R2, and RAB7A) were classified as risk-associated genes (HR > 1), while CTNNA1, PPP2CB, LRRC59, CAMTA1, and WIPI1 were regarded as protective genes (HR < 1). The CAFG risk score was calculated for each person by leveraging these genes. Subsequently, the TCGA-COAD, GSE17536, and GSE159216 cohorts were separated into low-risk and high-risk groups based on the median risk scores. Studies revealed that individuals belonging to the low-risk group showed better OS outcomes in comparison to those in the high-risk group (TCGA, HR = 3.272, 95% CI: 2.008-5.332, P < 0.001; GSE159216, HR = 1.924, 95% CI: 1.197-3.092, P = 0.031; GSE17536, HR = 1.496, 95% CI: 1.708-2.075, P = 0.005, **Figures 3D-F**). The distribution and scatter plot of CAFGs risk score showed that as risk score grew, OS declined while mortality increased (**Figures 3G-I**). The AUC of the 1-, 3-, and 5-year TCGA-COAD cohorts were 0.762, 0.796, and 0.852, respectively. In the GSE159216 cohort, the 1-, 3-, and 5-year AUCs were 0.660, 0.588, and 0.599. The AUC of the GSE17536 were 0.913, 0.635, and 0.587, respectively (**Figures 3J-L**). The CAFG signature model demonstrated an effective and dependable method for forecasting OS in COAD patients.

### 3.4 CAFGs signature is an independent prognostic indicator

**Figure 4:**
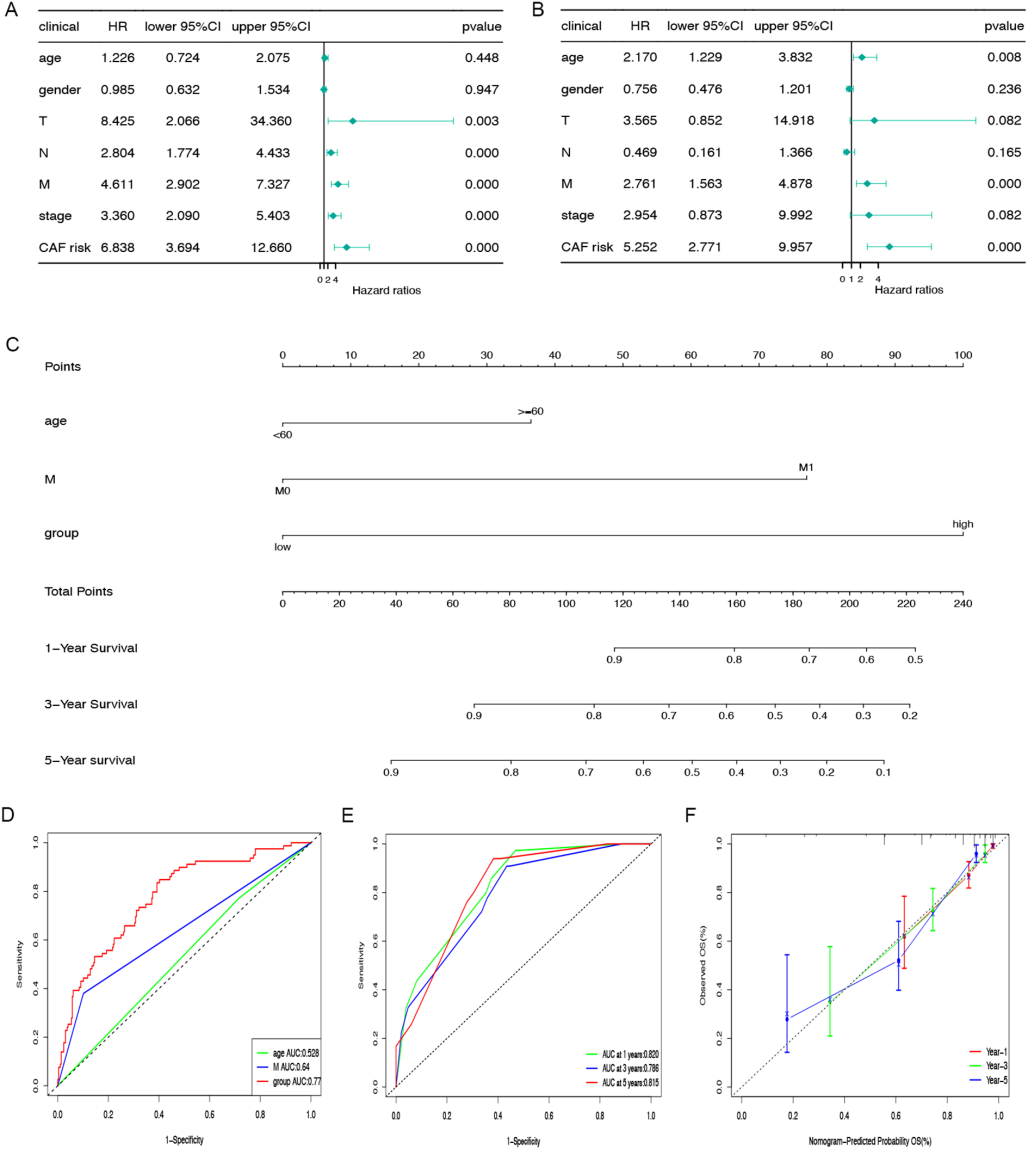
Nomogram construction and evaluation. The correlations between OS and CAFGs risk scores and other clinical indicators in TCGA-COAD populations were examined using univariate **(A)** and multivariate **(B)** Cox regression analyses. **(C)** The nomogram was applied to predict the 1-, 3-, and 5-year OS and the total score on the bottom scale implies the probability of OS. **(D)** ROC curves to evaluate the age, M stage and CAFGs risk group accuracy for predicting in patients. **(E)** ROC curves to evaluate the nomogram accuracy for predicting 1-, 3-, and 5-year OS in patients. **(F)** Calibration curves of the nomogram for predicting survival rates at 1-, 3-, and 5- years.

In the TCGA-COAD cohort, univariate and multivariate Cox regression analyses were performed to evaluate whether the prognostic relevance of CAFGs-associated gene signature was autonomous of factors like age, gender, and TNM stage. As shown in **Figures 4A, B**, both analyses confirmed that the CAFGs signature is an independent prognostic indicator. By utilizing the risk scores calculated by the Cox regression coefficients for the 11 genes associated with CAFs, combined with clinical attributes like age and M stage from the TCGA-COAD dataset, a nomogram was constructed to predict the 1-, 3-, and 5-year OS rates for COAD patients (**Figure 4C**). We assessed the discriminatory power of the nomogram using ROC and the AUC for the CAFGs risk grouping model was 0.77 (**Figure 4D**). To assess the reliability of the risk model, we calculated the area beneath the time-dependent ROC curve for OS. The AUC values were 0.820, 0.786, and 0.815 for the one-year, three-year, and five-year predictions (**Figure 4E**). The calibration curve demonstrated that the model’s OS predictions aligned with the dataset’s outcomes (**Figure 4F**).

### 3.5 Tumour immune infiltration

**Figure 5:**
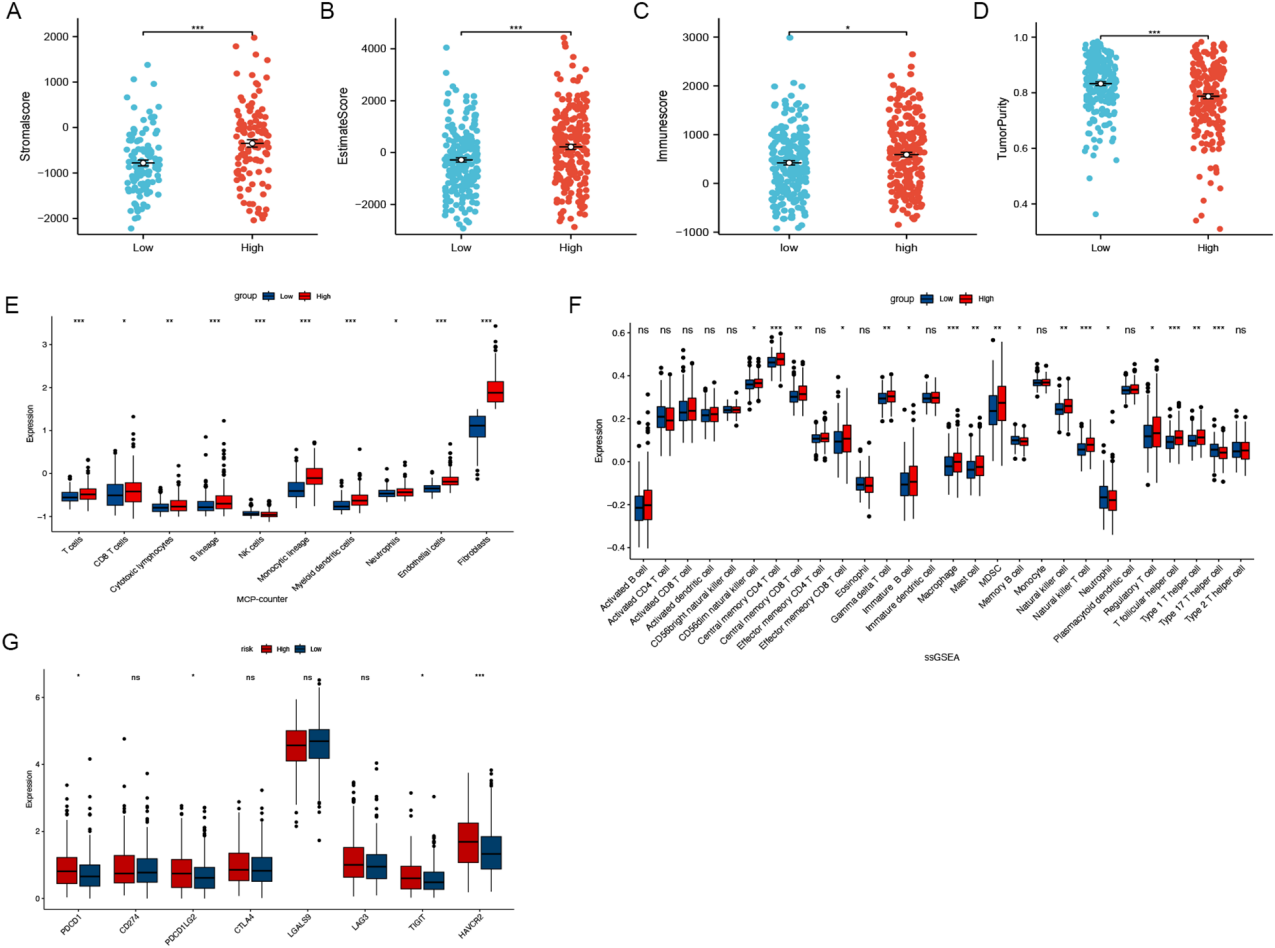
Immune infiltration analysis. **(A-D)** Different expression levels of stroma score, estimate score, immune score and tumour purity between the low- and high-risk groups. **(E)** The MCPcounter algorithm estimated the expression levels of ten different cell types, including fibroblasts. **(F)** The association of CAFGs risk score with 28 tumor-infiltrating immune cells. **(G)** Differential expression levels of the immune checkpoint-related genes between low- and high-risk groups. (ns, no significance, *P < 0.05, **P < 0.01, ***P < 0.001).

Differences in the expression levels of the stroma score, estimate score, immune score, and tumor purity were identified across the low-risk and high-risk groups (**Figures 5A-D**). Using the MCPcounter algorithm, the abundance of 10 cell categories, comprising eight immune cells, endothelial cells and fibroblasts, were compared between these two groups (**Figure 5E**), revealing a significantly higher prevalence of fibroblasts in the high-risk group. Additionally, results from the ssGSEA algorithm showed that central memory CD4+ T cells, NK cells, and macrophages showed higher expression in the high-risk group (**Figure 5F**). Given the importance of immune checkpoint inhibitors (ICIs) in immunotherapy, we analyzed the expression of eight common ICI-related genes in both groups. The analysis showed that PDCD1, PDCD1LG2, TIGIT, and HAVCR2 were more highly expressed in the high-risk group (**Figure 5G**). These results indicate that individuals in the high-risk group might be better candidates for ICI therapy.

### 3.6 Evaluation of somatic mutations and TMB analysis

**Figure 6:**
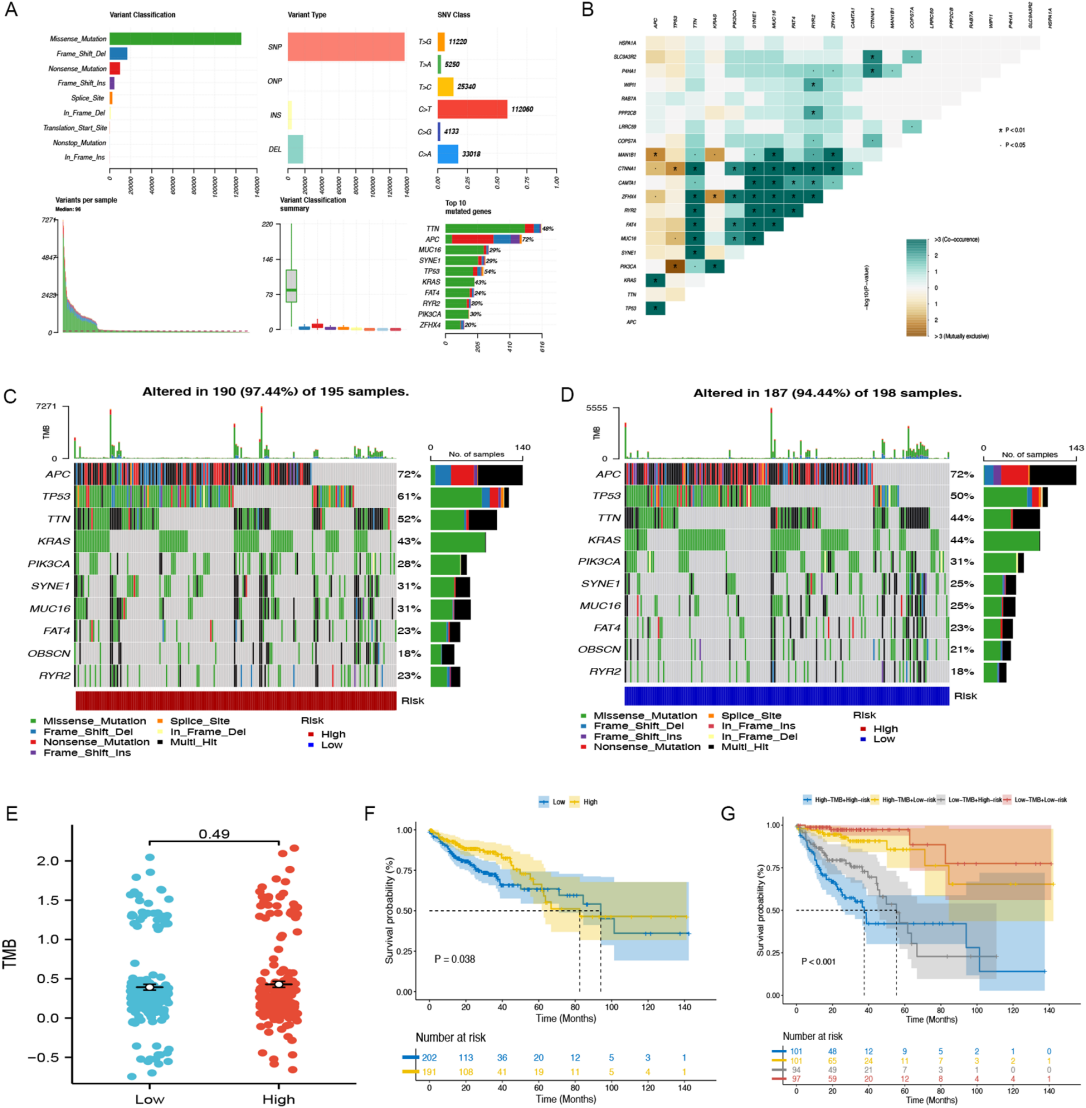
Somatic mutation in TCGA-COAD. **(A)** The general mutation profile. Different colors indicate different mutations. **(B)** Interaction relationship of major mutation genes. **(C)** The high-risk group’s gene mutation frequency. **(D)** The low-risk group’s gene mutation frequency. **(E)** Variations in TMB expression levels between groups. **(F)** The Kaplan-Meier curve between low- and high-TMB groups. **(G)** Kaplan-Meier analysis curves for patients categorized by TMB and CAFGs risk group.

**Figure 6A** illustrates the comprehensive genetic alteration landscape of COAD. In addition, somatic mutation interactions were detected, as shown in **Figure 6B**, where most genes had co-occurring mutations. In both the low-risk and high-risk categories, APC, TP53, and TTN emerged as the most commonly mutated genes (**Figures 6C, D**). Additionally, an analysis of TMB across groups revealed no substantial differences (P = 0.49) (**Figure 6E**). The K-M analysis indicated that the outcome of the low TMB group was better than that of the high TMB group (P=0.038) (**Figure 6F**). Notably, following the integration with our model, the outcome of the high-risk + high TMB group was considerably worse than that of the low-risk + low TMB group (**Figure 6G**).

### 3.7 Response to drug sensitivity predicted by CAFG signature

**Figure 7:**
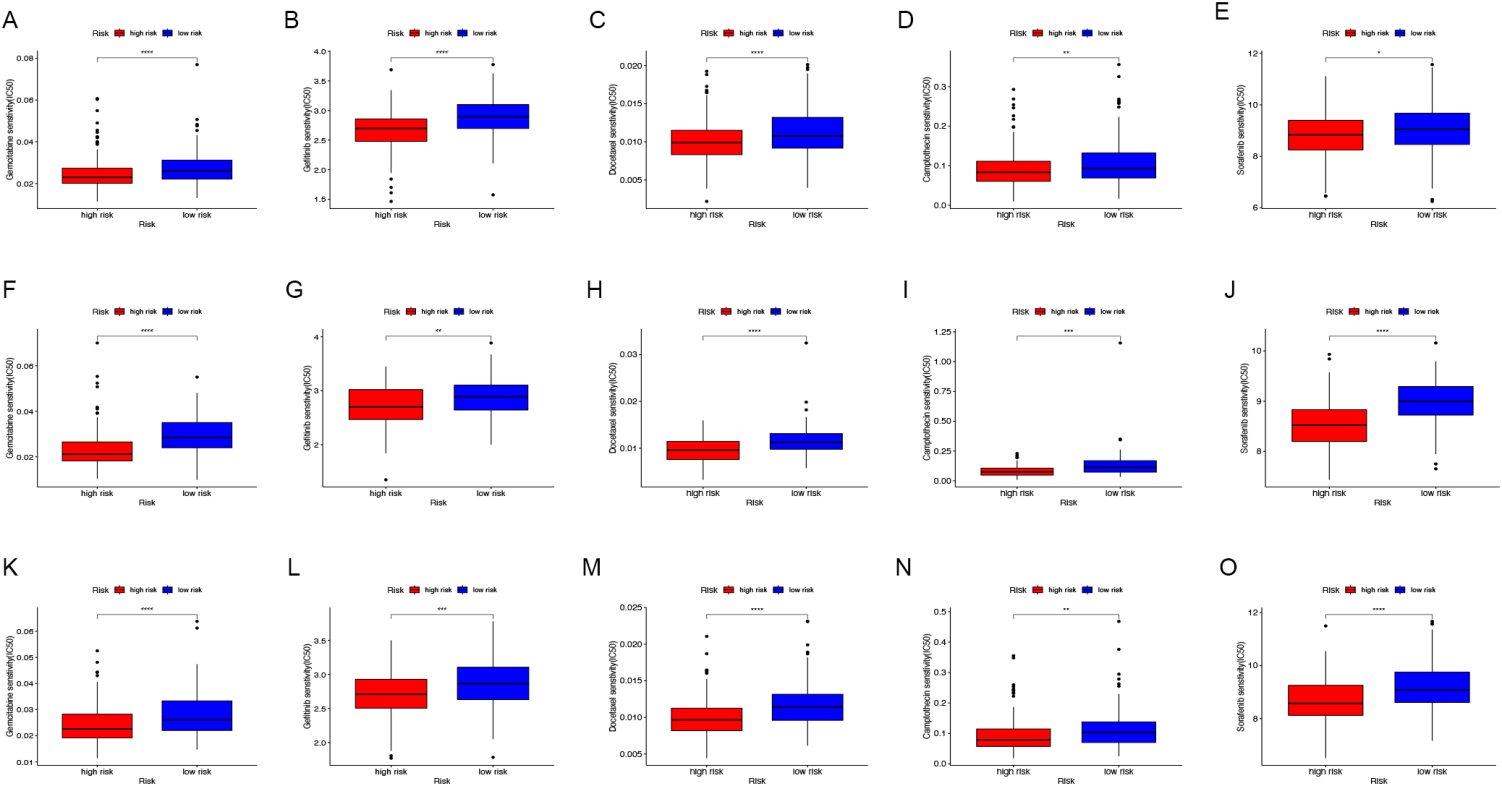
Drug sensitivity assessment. In the TCGA-COAD **(A-E)**, GSE17536 **(F-J)** and GSE159216 **(K-O)** cohorts, the IC50 values of Gemcitabine **(A, F, K)**, Gefitinib **(B, G, L)**, Docetaxel **(C, H, M)**, Camptothecin **(D, I, N)**, and Sorafenib **(E, J, O)** were compared between low-risk and high-risk groups. (*P < 0.05, **P < 0.01, ***P < 0.001, ****P < 0.0001).

Additionally, the differences in IC50 levels of chemotherapy drugs between the low-risk and high-risk groups in the TCGA-COAD cohort were investigated (**Figures 7A-E**). The analysis revealed that individuals in the low-risk category had higher IC50 values for anticancer drugs such as gemcitabine, gefitinib, docetaxel, camptothecin, and sorafenib. Comparable findings were noted in the GSE17536 (**Figures 7F-J**) and GSE159216 (**Figures 7K-O**) validation cohorts. These results indicate that the CAFG signature could be a useful predictor for selecting appropriate anticancer drugs in COAD treatment.

### 3.8 Tumor-suppressive effects of MAN1B1 knockdown

**Figure 8:**
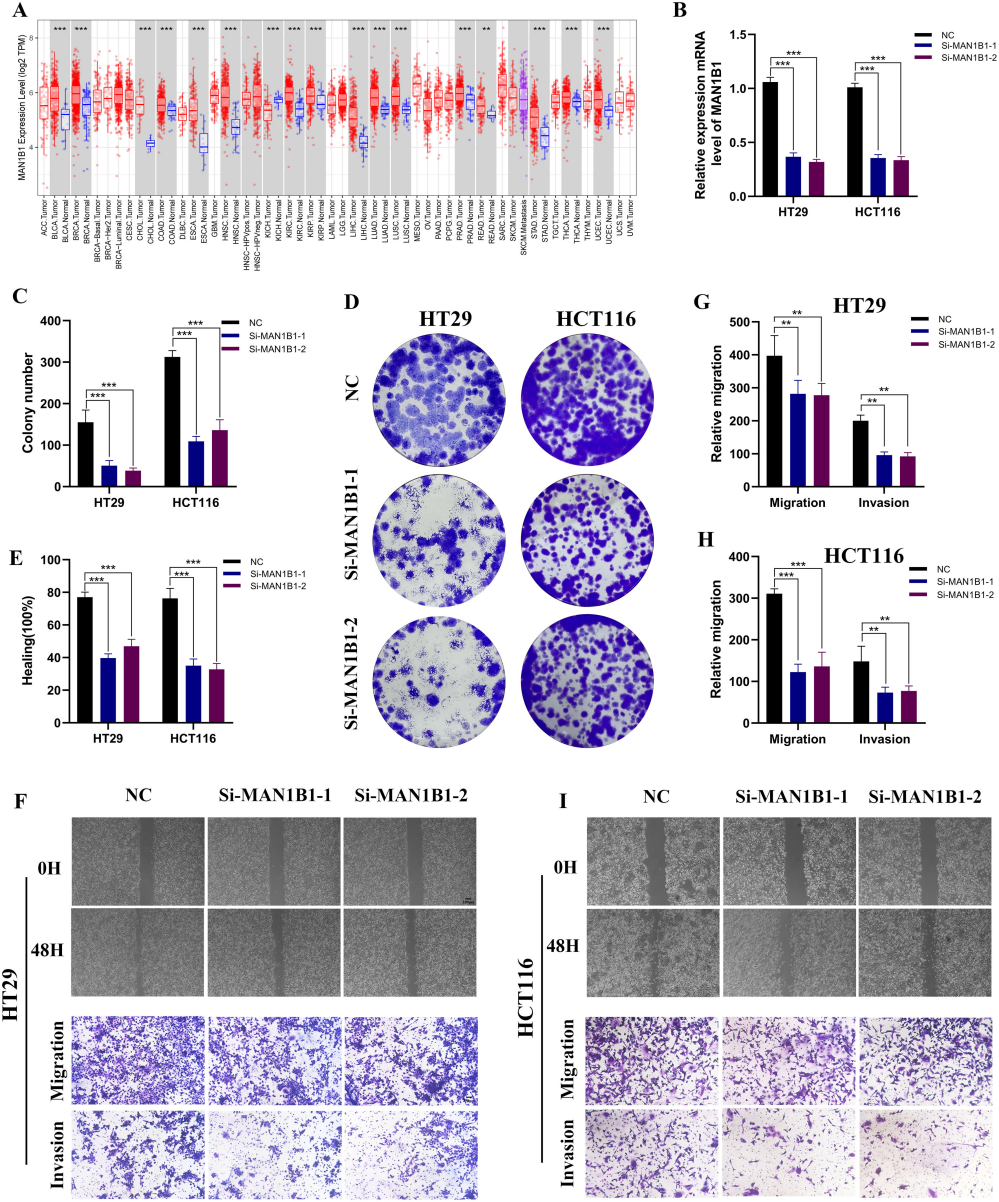
The impact of MAN1B1 in HCT116 and HT29. **(A)** The mRNA expression level of MAN1B1 in pan malignancies. **(B)** Following MAN1B1 knockdown, qRT-PCR showed a reduction in MAN1B1 expression. **(C, D)** As demonstrated by the cell colony formation experiment, cell proliferation was suppressed. **(E-I)** The capacity for invasion and migration dramatically reduced following the MAN1B1 knockdown. (**P < 0.01, ***P < 0.001).

**Figure 8A**, which examined MAN1B1 expression levels across different malignancies, demonstrates that MAN1B1 mRNA expression was considerably greater in COAD samples than in normal samples. We used siRNAs to knock down MAN1B1 expression in HCT116 and HT29 cells during *in vitro* experiments, and qRT-PCR confirmed this (**Figure 8B**). Colony formation experiments were conducted to evaluate the proliferative capacity of COAD cells. The findings indicated that the colony formation rate in the MAN1B1 knockdown group was significantly lower than the control group (**Figures 8C, D**). To additionally explore the impact of MAN1B1 on cell invasion and migration, transwell and wound healing assays were conducted. The data revealed that reducing MAN1B1 expression reduced cells’ invasion and migration capabilities (**Figures 8E-I**). Our results demonstrate that inhibiting MAN1B1 expression markedly suppresses COAD cell proliferation.

## 4 Discussion

CAFs constitute the most prevalent cell type in connective tissue, and their origin and function remain difficult to determine. Due to their phenotypic and functional heterogeneity, there is currently a lack of clear biomarkers (4, 20, 21). CAFs experience epigenetic modifications, releasing secretory factors that affect tumor angiogenesis, immune responses, and metabolism. Through intricate interactions with other cells, they actively contribute to tumor advancement (22, 23). Targeting specific CAF subtypes or converting CAFs into normal fibroblasts or anti-tumor phenotypes may offer therapeutic advantages for patients. However, in clinical practice, it is not always necessary to eliminate or reprogram CAFs. Blocking the signaling pathways from CAFs can also effectively achieve positive clinical outcomes. For example, targeting CXCL12 to antagonize the development of pancreatic cancer associated with FAP-expressing cancer-associated fibroblasts (24, 25). Nevertheless, the clinical application of CAFs in COAD presents challenges, prompting us to explore new CAF indicators. Analyzing the single-cell genome dataset, we identified a specific fibroblasts subset and developed a robust 11 CAFGs-related profile. The profile can predict prognosis, evaluate stromal components in the tumor microenvironment, and assess treatment responsiveness in COAD patients. Cox regression analyses established the CAFGs profile as an independent predictor of OS. To enhance its predictive precision and support clinical utility, we created and tested a nomogram incorporating age, M stage, and the CAFGs profile to forecast OS. The reliability of this model was confirmed through ROC and calibration curves, emphasizing its potential for clinical application. We also screened for chemotherapeutic drugs sensitive to high-risk populations, including gemcitabine, gefitinib, docetaxel, cephalexin, and sorafenib. These findings suggest that our model is reliable in predicting COAD prognosis and informing treatment decisions.

The tumor microenvironment and immunotherapy are crucial factors in the progression and treatment of COAD. Multiple studies have revealed the dynamic changes among various cell types and their interactions inside the COAD microenvironment through single-cell RNA sequencing and spatial transcriptomics technologies (26–29). Fibroblasts, as a key element of the tumor microenvironment, exhibit heterogeneity essential in regulating the tumor’s immune environment (30). Cancer-associated fibroblasts expressing MMP14 within the tumor immune microenvironment could be a promising therapeutic option in advancing stage III COAD (31). Our findings indicate that infiltration levels of immunosuppressive cells within the tumor tissues of high-risk COAD patients are significantly increased, including CD8 T cells, regulatory T cells, and tumor-associated macrophages. TAMs attract regulatory T cells (Tregs) by secreting the chemokine CCL2, establishing an immunosuppressive microenvironment in COAD (32, 33). Conversely, high-risk COAD patients have fewer NK cells in their tumor microenvironment. NK cells function as cytotoxic innate lymphocytes that kill tumor targets and coordinate immune responses through cytokines and chemokines (34, 35). Combining cetuximab with IL-2 and IL-15 boosts the cytotoxic activity of NK cells against COAD cell lines (36). We observed a notable increase in the expression of various immune checkpoint genes in the high-risk subgroup relative to the low-risk group, including PDCD1, PDCD1LG2 (PD-L1), TIGIT, and HAVCR2. The overexpression of PD-L1 can reduce the cytolytic function of T cells, thereby greatly enhancing tumor progression (37). The anti-PD-1/PD-L1 interaction has proven effective in COAD immunotherapy (38). Based on our findings, COAD patients in the high-risk group could be more suitable candidates for immune checkpoint blockade treatment.

MAN1B1, a newly identified tumor-associated gene, encodes a class I alpha-1,2-mannosidase. Alterations in this gene are known to result in autosomal-recessive intellectual disability (39–41). Studies suggest MAN1B1 as a potential cancer therapy target, promoting bladder cancer progression and linked to poor outcomes (42). Hepatitis B virus facilitates liver cancer development by increasing MAN1B1 expression (43). A separate study indicated that miR-125b regulates liver cancer formation by targeting the product of the MAN1B1 gene (44). MAN1B1 was identified as a harmful predictor, highly expressed in most malignant tumors. *In vitro* studies showed that MAN1B1 knockdown reduced COAD cell growth and colony formation. Furthermore, cell migration and invasion capabilities were significantly diminished. These results suggest that MAN1B1 may contribute to the onset and advancement of COAD. Nonetheless, this research has certain constraints. Further validation of the CAFGs signature in larger, independent clinical cohorts to confirm its prognostic accuracy and clinical utility. Additionally, mechanistic studies are needed, particularly its interactions with stromal and immune cells in the tumor microenvironment. Finally, exploring the combination of CAFGs-based therapies with existing treatments, such as immune checkpoint inhibitors, could provide synergistic benefits and improve outcomes for COAD patients.

## 5 Conclusion

In this research, we developed and confirmed a CAFGs-related signature, a prognostic marker for individuals with COAD. Additionally, we showcased MAN1B1’s role in colon adenocarcinoma via *in vitro* assays, suggesting its suitability as a potential target for COAD therapeutics. These results offer valuable insights for studies on anti-CAFs therapies, especially for individuals unresponsive to existing treatment strategies.

## Data Availability

The original contributions presented in the study are included in the article/**Supplementary Material**, further inquiries can be directed to the corresponding author/s.
