# Supplementary material for: Cancer-associated fibroblasts gene signature: a novel approach to survival prediction and immunotherapy guidance in colon cancer
Source: Front Immunol. 2025 Apr 8;16:1532306. doi: 10.3389/fimmu.2025.1532306 (PMC12011795; doi:10.3389/fimmu.2025.1532306)
Supplement: Supplementary file 1 [file Table1.docx]

| **Oligonucleotides** | **Nucleotide sequence (5'-3')** |
| --- | --- |
| **siRNA** |  |
| Scramble control | GCUUCGCGCCGUAGUCUUA |
| Si-MAN1B1-1 | CCTGAGAACTTACCTGAGATT |
| Si-MAN1B1-2 | GCTCAAGTATCTGTTCTTGCT |
|  |  |
| **Primer** |  |
| GAPDH | GGCCTCCAAGGAGTAAGACC (forward) |
|  | AGGGGAGATTCAGTGTGGTG (reverse) |
| MAN1B1 | TCACAGGGGACCGCAAATAC (forward) |
|  | TGAGCAGGTTTGGGTCATCG (reverse) |
|  |  |

**Table S2. Oligonucleotides used in research**
