# Supplementary figures and images for: Cancer-associated fibroblasts gene signature: a novel approach to survival prediction and immunotherapy guidance in colon cancer
Source: Front Immunol. 2025 Apr 8;16:1532306. doi: 10.3389/fimmu.2025.1532306 (PMC12011795; doi:10.3389/fimmu.2025.1532306)

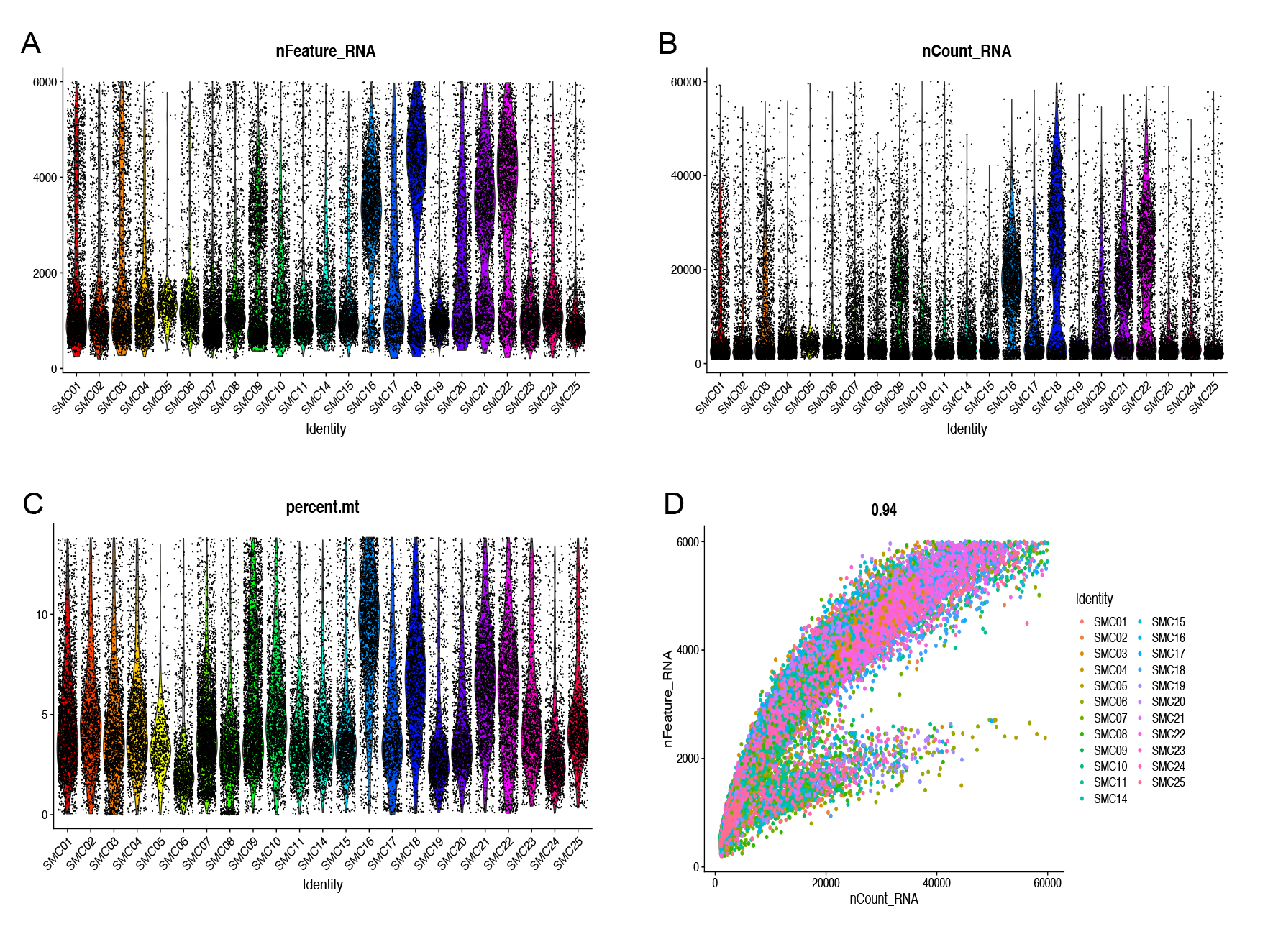

Supplement: Supplementary file 3 [file Image1.tif]
